# Workload Accommodations for Late Career Physicians: Associations Between Call Schedule and Disruptive Behavior

**DOI:** 10.1093/geroni/igaf122.4234

**Published:** 2025-12-31

**Authors:** Miranda McDaniel, Taylor Evans, Michael Williams, Betsy Williams

**Affiliations:** Professional Renewal Center, Lawrence, Kansas, United States; Professional Renewal Center, Lawrence, Kansas, United States; Professional Renewal Center/Wales Behavioral Assessment, Lawrence, Kansas, United States; Professional Renewal Center, Lawrence, Kansas, United States

## Abstract

The recent Federation of State Medical Board census data revealed a trend of increasing physician attrition1. To maximize retention of late career physicians, it will be important for healthcare systems to consider accommodations that will reduce physician stress and burnout and support maximal performance. Factors such as lack of sleep and workload contribute to feelings of burnout and can be associated with behavioral difficulties and interpersonal problems. 2. Especially for late career physicians, workload is recognized as a potential risk factor and impediment to staying in the workforce4. In a study examining senior emergency physicians, senior physicians noted increasing difficulties with their workload including difficulty recovering from overnight shifts3. Our study explores associations between physicians’ call schedules and disruptive workplace behavior across the career span. This study utilizes a sample of physicians between 23 and 84 years of age (N = 89, mean 56) referred for a comprehensive fitness for duty assessment. The findings of this study indicate late career physicians who were referred for a fitness for duty assessment secondary to disruptive behavior had greater call schedule workloads, there is a significant Age X Call Schedule interaction as well. This research has implications for how to retain and optimize the functioning of late career physicians. Additionally, this study has implications for increasing methods of age inclusivity in the workplace.

